# Positive childhood experiences and loneliness in medical students: the role of adaptive emotion regulation

**DOI:** 10.3389/fpsyg.2026.1809618

**Published:** 2026-04-07

**Authors:** Lauren Walkon, Michael Nazmifar, Tina Izad, Rachel Lloyd, Changiz Mohiyeddini

**Affiliations:** Oakland University William Beaumont School of Medicine, Rochester, MI, United States

**Keywords:** adaptive emotion regulation, loneliness, medical education, medical students, positive childhood experiences, psychological resilience, wellbeing

## Abstract

**Background:**

Loneliness is a highly prevalent issue among medical students, associated with anxiety, depression, and reduced academic performance. Developmental research suggests that positive childhood experiences (PCEs) may foster adaptive emotion regulation (ER) skills, which in turn buffer against loneliness. Relying on developmental psychopathology and emotion regulation frameworks, this study examined whether adaptive ER mediates the relationship between PCEs and loneliness.

**Methods:**

A cross-sectional survey was conducted with medical students (*N* = 97) at a U. S. medical school. Validated measures assessed PCEs, adaptive ER, and loneliness. Mediation was tested using bootstrapped regression models with 5,000 resamples.

**Results:**

Higher PCEs were significantly associated with greater use of adaptive ER strategies (*β* = 0.21, *p* < 0.001), which in turn were associated with lower loneliness (*β* = −0.19, *p* < 0.01). The indirect effect of PCEs on loneliness via adaptive ER was significant [indirect *β* = −0.039, 95% CI (−0.073, −0.013)], supporting the hypothesized mediation model.

**Conclusion:**

The findings support a theoretically grounded model linking developmental experiences to current wellbeing in medical students, with adaptive ER identified as a potential mechanism. However, given the cross-sectional design, causal inferences cannot be drawn. Results highlight adaptive ER as a modifiable intervention target for reducing loneliness, while PCEs highlight the importance of primary prevention efforts targeting children and families. Replication with larger, multi-site, and longitudinal studies is warranted.

## Introduction

Loneliness is characterized by a complex interplay between negative emotional states and unmet social needs. The disparity between one’s desired social relationships and one’s perception of existing relationships leads to loneliness. This underscores the uniqueness of loneliness, as it arises from perceived discrepancies between desired and actual social relationships (Perlman and Peplau, 1981).

For example, someone who enjoys solitude is not necessarily lonely, while an individual surrounded by others may still feel lonely. The difference lies in whether the person perceives their social needs as being met. Simply being surrounded by other people does not resolve feelings of loneliness; rather, meaningful and higher-quality relationships are required.

Furthermore, neurobiological evidence distinguishes loneliness from solitude by linking it to heightened activation in brain regions associated with social threat processing (e.g., the anterior cingulate cortex), highlighting its role as an indicator of unmet social needs (Cacioppo et al., 2016).

Loneliness can perpetuate a self-reinforcing cycle in which withdrawal exacerbates hypervigilance to social threats, further isolating the individual. Meta-analytic data confirm that loneliness is associated with elevated cortisol levels and inflammatory markers (Smith et al., 2020), underscoring its bidirectional relationship with physiological stress reactivity.

Accordingly, the more an individual isolates themselves, the more they approach social interactions with hypervigilance and negativity to protect themselves.

Recognizing the profound impact of loneliness on health outcomes, the World Health Organization has classified loneliness as a global public health concern since 2021 (Jaffe, 2023). The prevalence of loneliness has increased since 1979, with current estimates suggesting that nearly half of Americans experience loneliness (Bruss, 2024).

Negative childhood experiences (NCEs), a broad category encompassing childhood maltreatment, abuse, neglect, household dysfunction, and exposure to violence or parental mental illness, often act as a precursor to loneliness (Buecker et al., 2024). NCEs shape how individuals view relationships and can predispose them to social isolation. NCEs disrupt the development of prefrontal-amygdala circuitry, impairing the development of emotion regulation skills (Teicher and Samson, 2016).

Emotion regulation encompasses the cognitive and behavioral strategies used to modulate emotional experiences and responses (Gross, 1998). Research has shown that children exposed to negative childhood experiences tend to rely more heavily on maladaptive emotion regulation strategies (Kim and Cicchetti, 2010).

In contrast, positive childhood experiences (PCEs)—defined as experiences of safety, emotional support, belonging, and reliable caregiving during childhood, as assessed by instruments such as the Benevolent Childhood Experiences Scale (Narayan et al., 2018), enhance one’s capacity to form secure attachments and develop more positive interpersonal relationships, which in turn support adaptive emotion regulation strategies such as cognitive reappraisal.

PCEs are historical events that cannot be changed retrospectively and therefore cannot themselves serve as direct targets for intervention. However, identifying the psychological mechanisms through which PCEs exert their effects, specifically, adaptive emotion regulation, is of direct applied relevance because emotion regulation is a modifiable skill amenable to training. The contribution of the present study lies not in positioning PCEs as an intervention lever, but in clarifying that their protective effect on loneliness operates partly through a pathway that can be actively strengthened. Intervening on adaptive emotion regulation, therefore, represents a feasible strategy for reducing loneliness even among adults whose early childhood experiences were not optimal.

Empirical evidence indicates that both negative and positive childhood experiences are associated with distinct emotion regulation strategies. For instance, NCEs are associated with higher engagement in dysfunctional ER strategies, such as rumination, catastrophizing, and emotional suppression (Mohiyeddini et al., 2014; Mohiyeddini and Opacka-Juffry, 2015).

ER deficits, particularly rumination and suppression, are linked to loneliness through increased social withdrawal and reduced tendencies toward self-disclosure (Preece et al., 2021).

In contrast, PCEs help individuals engage in adaptive emotion regulation, enabling more deliberate cognitive appraisal and improving the ability to sustain social bonds (Narayan et al., 2018).

Meta-analytic evidence supports these mechanistic links. A recent systematic review and meta-analysis demonstrated that low levels of adaptive ER (particularly cognitive reappraisal) are strongly associated with higher loneliness across age groups (Preece et al., 2021). Similarly, another meta-analysis established that adaptive ER strategies buffer the negative effects of adverse childhood experiences on social connectedness (Miu et al., 2022).

Consequently, adaptive ER serves not only as a protective factor against loneliness but also as a foundational component of long-term psychological resilience. Within medical student populations specifically, research has documented elevated rates of loneliness and demonstrated that resilience factors, including supportive childhood relationships, buffer the negative impact of adverse experiences on wellness outcomes (Sciolla et al., 2019; Blickenstaff et al., 2022).

## Study hypotheses

Building on this literature, the present study proposes a conceptual model in which positive childhood experiences (PCEs) are associated with lower levels of loneliness, both directly and indirectly through adaptive emotion regulation strategies, which serve as a mediating mechanism.

Accordingly, we hypothesized that:

*H1*: Positive Childhood Experiences (PCEs), as measured by the Benevolent Childhood Experiences Scale, will be negatively associated with loneliness, as measured by the UCLA Loneliness Scale.

*H2*: PCEs will be positively associated with adaptive emotion regulation, as measured by the Cognitive Emotion Regulation Questionnaire (CERQ-Short).

*H3*: Adaptive emotion regulation will be negatively associated with loneliness.

*H4*: Adaptive emotion regulation will mediate the relationship between PCEs and loneliness.

## Materials and methods

### Study design

This study employed a cross-sectional, survey-based design to examine whether adaptive emotion regulation mediates the relationship between PCEs and loneliness among medical students.

A non-interventional, single-arm design was used to collect self-reported data on emotion regulation, loneliness, and PCEs at a single point in time.

### Power analysis

An *a priori* power analysis was conducted using G*Power* (Faul et al., 2009) *to estimate the required sample size. Because G*Power does not offer a dedicated mediation-specific procedure, we followed the approach recommended by Fritz and MacKinnon (2007) and used the linear multiple regression module (fixed model, R^2^ increase) to approximate the sample size needed to detect a small-to-moderate indirect effect. Assuming a standardized indirect effect equivalent to Cohen’s *f*^2^ = 0.10 (corresponding to a small-to-moderate effect), *α* = 0.05, and power = 0.95, the analysis indicated a required sample size between *N* = 79 and *N* = 111. The final sample of *N* = 97 students with complete data falls within this range, indicating that the study was adequately powered to detect the hypothesized indirect effect. We acknowledge that d-based estimates are not the most direct metric for mediation; future studies should supplement this approach with Monte Carlo power simulations using mediation-specific software such as the MBESS package in R (Kelley, 2007) or the online tool by Schoemann et al. (2017), which allows power estimation directly from the indirect effect and its standard error.

### Sampling and procedure

Participants were recruited from all 4 years of study at Oakland University William Beaumont School of Medicine (OUWB) through institutional email invitations.

Institutional email invitations were distributed to all enrolled students across all 4 years of the OUWB medical program. At the time of data collection, the total enrolled student body comprised approximately 640 students. Of these, 110 students responded to the survey, yielding an approximate response rate of 17%. Thirteen participants were excluded due to incomplete responses (listwise deletion), resulting in a final analytic sample of *N* = 97. Among these, 60.8% identified as female, 39.2% as male, and the mean age was 24.67 years (SD = 1.71). Students were distributed across all four years: M1 (13.6%), M2 (57.3%), M3 (11.8%), and M4 (8.2%), with the remaining 9.1% not specifying their year. The predominance of M2 students likely reflects the timing of the survey distribution, which fell during a period of peak M2 engagement with the curriculum.

Participation was voluntary, and no compensation was provided.

The survey remained open for 7 months (October 2023–April 2024) to allow adequate participation, with reminder emails sent periodically.

### Measures

All measures used in this study have been previously validated and demonstrate strong psychometric properties in similar populations.

Loneliness: Loneliness was assessed using the UCLA Loneliness Scale (Version 3) (Russell, 1996), a 20-item measure rated on a 4-point scale from 1 (never) to 4 (often). Higher scores indicate greater loneliness. In the present sample, Cronbach’s *α* = 0.92.

Adaptive Emotion Regulation: Adaptive emotion regulation was measured using the Cognitive Emotion Regulation Questionnaire-Short (CERQ-Short) (Garnefski and Kraaij, 2006), which assesses 9 cognitive emotion regulation strategies. Following prior research (Miu et al., 2022), we computed an adaptive ER composite score by summing the subscale scores for acceptance, positive refocusing, refocus on planning, positive reappraisal, and putting into perspective. Higher scores indicate greater use of adaptive strategies. Cronbach’s *α* = 0.88.

Positive Childhood Experiences: PCEs were assessed using the Benevolent Childhood Experiences Scale (Narayan et al., 2018), a 10-item measure that asked participants to indicate whether they experienced specific positive childhood experiences (e.g., “Had at least one caregiver with whom you felt safe”). Scores range from 0 to 10, with higher scores indicating more PCEs. Cronbach’s α = 0.79.

## Results

Descriptive statistics were calculated for the primary study variables: loneliness, adaptive emotion regulation, and PCEs. The mean score on the UCLA Loneliness Scale was *M* = 1.95 (SD = 0.52), indicating moderate levels of loneliness in the sample. The mean score for adaptive emotion regulation was *M* = 3.48 (SD = 0.84), suggesting a moderate to high tendency to use adaptive strategies. The mean score for PCEs was *M* = 8.68 (SD = 2.24), indicating relatively high levels of positive childhood experiences.

Age was negatively correlated with PCEs (*r* = −0.35, *p* < 0.01), suggesting that older students reported slightly lower levels of positive childhood experiences. Gender was negatively associated with adaptive ER (*r* = −0.22, *p* < 0.05), suggesting that male students reported lower levels of adaptive emotion regulation strategies.

The correlations among loneliness, adaptive ER, and PCEs were statistically significant (rs ≤ 0.59, *p* < 0.01), supporting Hypotheses 1–3.

### Mediation analysis

**Figure 1:**
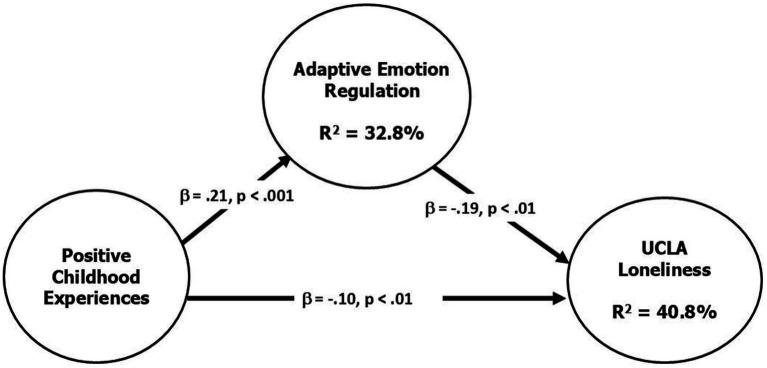
Results of the tested mediation model. Adaptive emotion regulation as a mediator between positive childhood experiences and loneliness. Standardized regression coefficients (*β*) are shown for the path from PCEs to adaptive emotion regulation (a path), the path from adaptive emotion regulation to loneliness (b path), and the direct path from PCEs to loneliness (c’ path). The indirect effect (a × b) was statistically significant [*β* = −0.04, 95% CI (−0.073, −0.013)].

A hierarchical linear regression analysis was conducted to investigate the proposed mediation model. Age and gender were entered in the first step, followed by adaptive emotion regulation and PCEs. Figure 1 presents the results of the tested mediation model, with standardized coefficients shown for each pathway.

### Association between PCEs and adaptive emotion regulation

Positive childhood experiences significantly predicted greater use of adaptive emotion regulation strategies [*β* = 0.21, *b* = 0.21, SE = 0.03, *t*(92) = 6.70, *p* < 0.001]. The model explained 32.8% of the variance in adaptive emotion regulation [*R*^2^ = 0.33, *F*(1, 92) = 44.93, *p* < 0.001], indicating a strong association between PCEs and emotion regulation capacity.

### Mediation of the PCEs-loneliness relationship

When including both PCEs and adaptive emotion regulation as predictors of loneliness, the full model accounted for 40.8% of the variance in loneliness scores [*R*^2^ = 0.41, *F*(2, 91) = 31.29, *p* < 0.001].

Adaptive emotion regulation was negatively associated with loneliness [*β* = −0.19, *b* = −0.19, SE = 0.06, *t*(91) = −3.00, *p* = 0.004], while PCEs continued to exert a significant direct effect [*β* = −0.10, *b* = −0.10, SE = 0.02, *t*(91) = −4.28, *p* < 0.001].

### Indirect effect

The indirect effect of PCEs on loneliness through adaptive emotion regulation was statistically significant [*β* = −0.04, *b* = −0.04, SE = 0.01, 95% CI (−0.073, −0.013)]. This finding suggests that part of the protective association between PCEs and loneliness operates through greater use of adaptive emotion regulation strategies.

## Discussion

The present study examined whether adaptive emotion regulation mediates the association between positive childhood experiences (PCEs) and loneliness among medical students. Three key findings emerged. First, higher PCEs were significantly associated with greater use of adaptive emotion regulation strategies. Second, greater use of adaptive emotion regulation was associated with lower loneliness. Third, and most centrally, adaptive emotion regulation functioned as a significant partial mediator of the PCE–loneliness association [indirect *β* = −0.04, 95% CI (−0.073, −0.013)], accounting for approximately 15% of the total effect. Taken together, these findings support the hypothesized developmental model in which early positive relational experiences shape emotion regulatory capacity, which in turn reduces vulnerability to loneliness in adulthood.

These findings align with prior research linking PCEs to higher use of adaptive emotion regulation strategies and lower loneliness in young adult populations (Narayan et al., 2018; Holman et al., 2021; Smith et al., 2024). For instance, Holman et al. (2021) found that medical students who reported higher levels of perceived social support—often rooted in earlier positive relationships—also reported lower loneliness. Similarly, prior work has shown that adaptive emotion regulation strategies, particularly cognitive reappraisal, mediate the association between supportive early environments and better mental health outcomes in university students (Miu et al., 2022). The present study extends this evidence specifically to a medical student population, suggesting that these developmental factors continue to shape wellbeing in the high-stress context of medical education.

The indirect effect size, while small in absolute terms (*β* = −0.04), is consistent with mediation effects typically observed in developmental and health psychology research (Kenny et al., 2014), and represents a theoretically meaningful pathway: approximately 15% of the total protective association between PCEs and loneliness is explained by adaptive emotion regulation alone.

It is essential to interpret the mediation findings with appropriate caution. While the statistical model is consistent with the proposed temporal ordering (PCEs → adaptive ER → loneliness), consistency with a causal model is not equivalent to demonstrating causation. The cross-sectional design means that temporal precedence cannot be established: it remains equally plausible that current loneliness influences how individuals regulate their emotions, or that loneliness colors the retrospective recall of childhood experiences, rather than the reverse. The mediation analysis identifies a statistically significant indirect pathway, but this should be understood as a cross-sectional association that is theoretically coherent with a developmental model—not as evidence that PCEs causally produce adaptive emotion regulation, which in turn causally reduces loneliness. Longitudinal studies with prospective measurement of all three constructs are required before causal conclusions can be drawn.

From a practical standpoint, these findings suggest that embedding structured emotion regulation training within existing medical school wellness curricula may help reduce loneliness. Evidence-based approaches such as mindfulness-based stress reduction, cognitive reappraisal training, and social–emotional skills workshops have been shown to improve adaptive emotion regulation and social connectedness in young adults (Galante et al., 2018; Denny et al., 2004). Offering such programs in small-group formats, integrated into pre-clinical coursework, may enhance feasibility and uptake while simultaneously fostering peer support networks. However, given the cross-sectional nature of the present data, such implications remain preliminary and should be tested through intervention trials with longitudinal follow-up.

## Limitations and future directions

This present study poses several limitations. First, although we used validated instruments, the reliance on self-report measures introduces the possibility of common method variance, potentially inflating associations between the variables due to shared measurement context.

Second, although our hypothesized model is grounded in developmental theory and prior longitudinal research findings, the cross-sectional design of the study means that temporal sequencing among PCEs, adaptive ER, and loneliness cannot be confirmed (Maxwell and Cole, 2007). Hence, causal inferences cannot be inferred from our data. Longitudinal or experimental studies are needed to determine whether PCEs prospectively influence adaptive ER skills and whether those skills, in turn, reduce loneliness. In addition, bidirectional relationships must be investigated in longitudinal studies (Little, 2013).

Third, the voluntary participation may have attracted students with a stronger interest in wellbeing or psychological research, potentially inflating average adaptive ER scores and underestimating loneliness prevalence relative to the broader medical student population. In addition, all variables were measured via self-report, which may be subject to recall bias and social desirability effects (Podsakoff et al., 2003). For instance, participants might overreport PCEs or adaptive ER in order to depict themselves more favorably. Future studies could, in addition to well-validated measurements that we used, incorporate objective data such as peer reports, behavioral tasks, or longitudinal diary methods to reduce potential bias and increase the internal validity of the results (Paulhus, 1991).

Fourth, the study did not measure several variables that may function as confounders of the observed associations. Depression and anxiety, for example, are both associated with reduced adaptive emotion regulation and elevated loneliness (Beutel et al., 2017), and may independently account for part of the observed PCE–loneliness association. Chronic academic stress, which is pervasive in medical education, could similarly suppress emotion regulation capacity and increase loneliness independently of childhood experiences. Social support is another important omitted variable: higher perceived social support has been shown to both enhance emotion regulation skills and reduce loneliness independently of developmental history (Holt-Lunstad et al., 2015), and its exclusion means that the mediation pathway may be partially confounded. Substance use, which is elevated in medical student populations and associated with both emotional dysregulation and social withdrawal, represents a further potential confounder. Without measuring and statistically controlling for these variables, we cannot rule out the possibility that the observed indirect effect is partly explained by unmeasured third variables. Future studies should incorporate comprehensive assessments of mental health symptoms, stress, social support, and health behaviors alongside the core constructs tested here.

Fifth, it is possible that loneliness could influence both ER tendencies and retrospective reports of PCEs, as the cross-sectional nature of our design raises the possibility of reverse causation.

Given the reliance on self-reported childhood experiences, recall bias may also distort associations, particularly if current affective states color memories of early life.

Finally, the sample was drawn from a single U. S. medical school, which may limit the generalizability of findings to other educational and cultural contexts. Cultural norms surrounding emotional expression, help-seeking behaviors, and social connectedness may shape both loneliness experiences and emotion regulation strategies (Zhang and Dong, 2022; Caldarelli et al., 2026). Additionally, institutional factors such as curriculum structure, class size, and availability of mental health resources may influence student wellbeing in ways not captured here. Recent cross-cultural research on university students in Mediterranean countries (Troncone et al., 2026; Caldarelli et al., 2026) highlights the importance of examining how cultural context moderates relationships between developmental experiences, coping strategies, and mental health outcomes. Future research should therefore examine these associations across diverse institutions, including medical schools with different pedagogical approaches, as well as across cultural contexts where the meaning and expression of loneliness may differ. Replication in larger, multi-site, and cross-cultural samples would further strengthen confidence in the robustness of these findings (Fritz and MacKinnon, 2007; Schoemann et al., 2017).

A path to translate these findings into preventive work would be if medical schools consider.

embedding structured ER training within existing wellness curricula. Evidence-based approaches such as mindfulness-based stress reduction, cognitive reappraisal training, and social–emotional Skills workshops have been shown to improve adaptive ER and social connectedness in young adults (Galante et al., 2018; Denny et al., 2004). Offering such programs in small-group formats, integrated into pre-clinical coursework, may enhance feasibility and uptake while fostering peer support networks. These efforts should be accompanied by longitudinal follow-up studies to determine whether improvements in emotion regulation translate into sustained reductions in loneliness.

## Conclusion

This study contributes to the growing wealth of knowledge under the umbrella of adaptive emotion regulation and its role in mitigating the effects of loneliness. Our statistically significant findings support the notion that emotion regulation is one of several mechanisms through which PCEs are associated with loneliness.

These results highlight the importance of considering both physiological and emotional dimensions of student wellbeing in an academic setting. In demanding educational contexts, such as medical education, addressing loneliness and strengthening adaptive emotion regulation strategies may offer a dual-pathway approach to enhancing students’ wellbeing and academic performance.

The findings support the notion that emotion regulation represents one mechanism through which positive childhood experiences are associated with lower loneliness, though causal confirmation requires longitudinal designs. On the basis of these findings, we aim to promote a scalable, evidence-based pathway to improve emotional resilience and wellbeing in the future healthcare professionals.

## Data Availability

The datasets presented in this article are not readily available because this study contains academic performance data of students that cannot be shared with any third party. Requests to access the datasets should be directed to mohiyeddini@oakland.edu.
